# Relationships among inferiority feelings, fear of negative evaluation, and social anxiety in Chinese junior high school students

**DOI:** 10.3389/fpsyg.2022.1015477

**Published:** 2023-01-10

**Authors:** Jiajian Li, Shuxin Jia, Lishen Wang, Mingming Zhang, Shunsen Chen

**Affiliations:** ^1^School of Educational Science and Fujian Key Laboratory of Applied Cognition & Personality, Minnan Normal University, Zhangzhou, China; ^2^Research Center of Brain and Cognitive Neuroscience, Liaoning Normal University, Dalian, China; ^3^College of Teacher Education, Shaoguan University, Shaoguan, China

**Keywords:** junior high school students, inferiority feelings, social anxiety, fear of negative evaluation, mediating effect

## Abstract

**Introduction:**

This study aimed to explore the relationship between feelings of inferiority and social anxiety in Chinese junior high school students. In addition, it examined the potential mediating effect of fear of negative evaluation in this relationship.

**Methods:**

A survey was administered to a sample of 734 Chinese junior high school students. The Feelings of Inadequacy Scale, Brief Fear of Negative Evaluation Scale, and Social Avoidance Distress Scale were used.

**Results:**

First, there were significant positive correlations between all subscales for the inferiority feelings, social anxiety, and fear of negative evaluation. Furthermore, fear of negative evaluation mediated the predictive effects of four inferiority subscales (i.e., self-esteem, academic ability, appearance, and physical ability) for social anxiety. However, the total score for the sense of inferiority and social confidence subscale lacked this mediating effect.

**Conclusion:**

The inferiority feelings of self-esteem, academic ability, appearance, and physical ability may directly and indirectly predict social anxiety through fear of negative evaluation.

## 1. Introduction

Junior high school students are in an important developmental transition period to maturity. During adolescence, the students facing psychophysical changes are vulnerable to psychological problems (Li et al., 2020). Although they are growing quickly, mental health services in China still have a lot of issues. Due to widespread stigma, a lack of human resources, and disjointed service delivery models, mental health services are underutilized in China (Liu et al., 2018b). Therefore, Chinese adolescents still face various psychological crisis and behavioral problems (Zhou et al., 2020), such as anxiety (Wu et al., 2021), depression (Wu et al., 2021), internet addiction (Chi et al., 2020), suicide (Li et al., 2021). In addition, students are under tremendous pressure from study and competition in China’s education system (Sun et al., 2012). A competitive education system in traditional Chinese culture has been linked with the high incidence of anxiety among adolescents (Liu et al., 2018a). In sociocultural situations with closely knit social networks, worries about being rejected and losing vital social resources may be stoked (e.g., East Asian cultures; Schunk et al., 2022). Chinese people may be higher in rejection avoidance than western. In this case, Chinese adolescents are probably more likely to be fearful of negative evaluations from peers or teachers. Some studies have shown that the East Asian prefer to avoid accrual of negative reputation (Yamagishi et al., 2008). In other words, Chinese adolescents tend to avoid social when they are concerned about negative perceptions of themselves. Therefore, it is very meaningful to study the psychological characteristics of junior high school students’ inferiority feelings, fear of negative evaluation and social anxiety.

Social anxiety is a common adolescent anxiety characterized by an unreasonable fear of negative appraisal in social situations (Morrison and Heimberg, 2013). Social anxiety is diagnosed when this concern begins to impair interpersonal communication. Compared with students in other age groups, junior high school students have higher levels of social anxiety. Furthermore, the incidence of social anxiety disorder in adolescence exceeds 50% (Aderka et al., 2012). Moreover, social anxiety increases risk for other clinical disorders such as depression (Kalin, 2020). Although social anxiety may be relieved with school closure during the COVID-19 outbreak, such improvement is likely to be short-lived (Morrissette, 2021). Children and youths with the high social anxiety traits will face significant challenges when schools reopen. Therefore, sustained and effective interventions are needed based on a full understanding of the causes of adolescent social anxiety during a pandemic.

Feelings of inferiority involves feelings of weakness and inability to help oneself (Ergun-Basak and Aydin, 2019). People with low self-esteem tend to despise themselves and believe that they are less valuable than others (Adler, 1927). Negative emotional experiences stem from underestimation of self in social comparisons. Severe feelings of inferiority are psychological defect (Adler, 1927). There are two core concepts for inferiority feelings: poor self-evaluation and negative emotional experience (Tang, 2012). The psychological development stage of junior high school students occurs during the adolescent period. Their self-awareness develops rapidly but inconsistently (Agbaria et al., 2012). They are very sensitive to the evaluations of others, especially negative ones. Thus, they cannot accurately perceive themselves, which may lead to low self-concept and even a sense of inferiority. People with high inferiority feelings are afraid to interact with others for fear of rejection and tend to adopt an avoidant approach, which may exacerbate social anxiety (Shim et al., 2013). Numerous researchers have also found that lower self-evaluation influences the development of social anxiety, and that lower self-evaluation is a major cause of inferiority feelings (Lin and Fan, 2022). According to the conceptual study of inferiority complex and social anxiety, inferiority complex is an individual’s negative evaluation of self, and the negative feedback from the external environment can lead to social anxiety. Studies have shown that the feelings of inferiority influenced social interactions and social relationships with peers (Collins, 1996; Wilkinson, 1999; Liu et al., 2022). In addition, inferiority feelings still explain a significant amount of variation in interpersonal rumination (Cimsir, 2019), and self-esteem is an important negative predictor of social anxiety (Yücens and Üzer, 2018; You et al., 2019). The current researches on inferiority have focused on the definition, cause, and solution (Lyu, 2022). However, individuals are often unique, and there is little discussion on commonality. Current limitations are that little research has focused on inferiority at specific levels, such as the inferiority of academics, appearance, social interaction, and physical ability. As a result, we proposed Hypothesis 1.

*Hypothesis 1 (H1)*: All subscales for the inferiority feelings are correlated with social anxiety.

**Figure 1:**
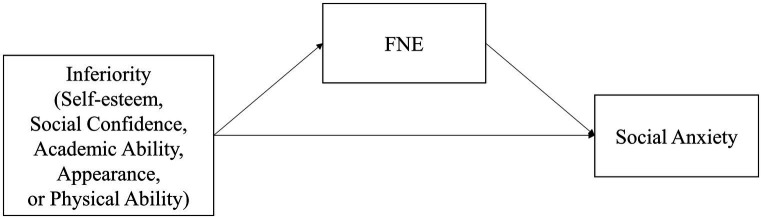
Hypothesized research model. FNE = fear of negative evaluation.

Watson and Friend (1969) defined fear of negative evaluation as apprehension about others’ appraisal, distress about the possibility of unfavorable judgment, avoidance of situations involving evaluations, and anticipation one would be unfavorably evaluated. The essence of inferiority feelings is the low self-esteem, and the individuals are unable to face their strengths and weaknesses in a rational and objective manner. Junior high school students in the sensitive period are still in the immature stage of self-awareness, which are prone to poor self-perception bias (Portillo and Fernández-Baena, 2019). “Biased self-perception” refers to the tendency to perceive one’s social performance as more negative than that of the observer, which is a characteristic of high fear of negatively evaluating individuals (Nordahl et al., 2017). Previous research has shown that individuals with lower self-esteem were more prone to have a higher level of fear of negative evaluation (Ahadzadeh et al., 2018). Due to poor self-representation and negative schemas, unpleasant experiences often accompany fear of evaluation. Inferiority feelings are therefore likely to contribute to the fear of negative evaluation. At the same time, fear of negative evaluation is considered as a cognitive and emotional risk factor for social anxiety (Haikal and Hong, 2010). It is closely related to trait anxiety and social avoidance (Stein et al., 2002). Throughout the literature, it has been found that fear of negative evaluations is associated with social anxiety (Zhong and Zhang, 2011; Cheng and Binrong, 2016). Some cognitive models suggest that social anxiety stems in part from fear of negative evaluations and excessive self-focus (Clark and Wells, 1995; Rapee and Heimberg, 1997). Excessive self-focus has some similarities with the characteristic egocentricity of inferiority feelings. Therefore, we proposed Hypothesis 2 and hypothesized research model (see Figure 1).

*Hypothesis 2 (H2)*: Fear of negative evaluation plays a mediating role between the subscales of inferiority and social anxiety.

Previous research has revealed a significant relationship between junior high school students’ inferiority feelings and social anxiety. However, few studies have explored the relationship between a specific level of inferiority and social anxiety, and the role of fear of negative evaluations in this relationship. Therefore, this study used a questionnaire method to investigate the relationship among these variables and aimed to provide a better theoretical basis to guide early prevention and treatment.

## 2. Materials and methods

### 2.1. Participants

The data were collected for over 20 days between 1 January 2019 and 20 January 2019. This research adopted convenience sampling to recruit 900 students from four public junior high school in Shaoguan, China as participants. Before investigation, we obtained informed consent from students and parents, and the investigators introduced the aims and procedures of this study to the students and assured confidentiality upon receipt of the questionnaire. The participants were given paper-based anonymous questionnaires in the classroom. The students of Grade 7 and Grade 8 were each selected from 7 classes for testing. Since the students of Grade 9 were facing the entrance examination, only 4 classes were selected. A total of 850 questionnaires were collected using group testing with immediate return. After discarding the invalid questionnaires (missing data >5%), 734 valid questionnaires were retained, and the effective rate was 86.4%. The students signed a written informed consent form before participating in this survey, and all their legal guardians agreed. This study was approved by the Ethics Committee of Minnan Normal University.

### 2.2. Measures

#### 2.2.1. Feelings of inferiority

This study used the Feeling of Inadequacy Scale (FIS; Fleming and Courtney, 1984) to assess the inferiority feelings. The FIS has a total of 36 items, including five dimensions. It measures the individual’s feelings of inferiority in terms of self-esteem, social confidence, academic ability, appearance, and physical ability. In the FIS, items 3, 6, 25, and 31 are scored in reverse. A 5-point Likert scale (1 = *never*, 5 = *always*) is used. The higher the score, the stronger the individual’s feelings of inferiority. In the present study, Cronbach’s α for the total FIS scale was 0.92.

#### 2.2.2. Social anxiety

The Chinese version of Social Avoidance and Distress Scale (SAD) was used in the current study (Wang et al., 1999). The SAD includes two factors: social avoidance and social distress. The former factor refers to the behavioral tendency to avoid social interaction. The latter represents feelings elicited in the situation. There are 14-item for each subscale. In the SAD, items 1, 3, 4, 6, 7, 9, 12, 15, 17, 19, 22, 25, 27, and 28 are scored in reverse. The scoring method uses *yes/no* responses. People with higher scores on the SAD scale are more anxious in actual interactions, and vice versa. In the present study, Cronbach’s α for the SAD was 0.86.

#### 2.2.3. Fear of negative evaluation

The study also used the Chinese version of Brief Fear Negative Evaluation Scale (BFNE; Leary, 1983; Chen, 2002). It consists of 12 items, using a 5-point Likert scale (1 = *completely inconsistent* and 5 = *extremely consistent*). In the BFNE, items 2, 4, 7, and 10 are scored in reverse. Cronbach’s α for the BFNE was 0.80 in the present study.

### 2.3. Data analysis

SPSS Version 22.0 (IBM, NY, United States) was used to conduct reliability analysis, the common method bias test, and Pearson correlation analysis. Finally, mediation analysis was conducted using the SPSS plugin PROCESS (Hayes, 2017). Bootstrapping was performed with 5,000 resamples and a confidence interval of 95%. The mediation analysis was considered significant when zero did not appear in the 95% confidence interval.

## 3. Results

### 3.1. Common method bias test

In order to reduce the common method bias brought by the self-reported questionnaire method, this study carried out procedural control by emphasizing anonymity and confidentiality during the data collection process. For checking the effectiveness of program control, we conducted a common method bias test (Podsakoff et al., 2003; Tang and Wen, 2020). The unrotated Harman’s single factor test result showed that there were 16 factors having the eigenvalue higher than 1. The explained variance of the first factor was 19.74%. This value was far below the critical level of 40%. Thus, the problem of common method bias in this study was not serious.

### 3.2. Descriptive statistics and correlations

This study finally selected 734 students as samples comprising 343 males (46.73%) and 391 females (53.27%), all aged 12–16 years old. The sample was made up of 260 Grade 7 students (35.42%), 307 Grade 8 students (41.83%) and 167 Grade 9 students (22.75%). In terms of family residence, 474 students (64.58%) lived in urban areas, while the remaining 35.42% resided in rural areas (n = 260). Regarding the issue of being an only child, 207 students (28.10%) were only children, while the remaining 527 students (71.80%) were not. As for parenting style, it was reported harsh and tough (23.85%) for 175 students, democratic and respectful (71.25%) for 523 students, and negligent and permissive (4.90%) for 36 students. Finally, self-assessment of students’ academic performance was based on five categories, such as excellent (9.95%), good (21.66%), average (36.37%), fair (23.98%), and poor (8.04%). The sample characteristics are shown in Table 1.

**Table 1:** Sample characteristics.

Characteristics	Options	Frequency	Percentage (%)
Age	12	70	9.54
	13	267	36.38
	14	249	33.92
	15	119	16.21
	16	29	3.95
Gender	Male	343	46.73
	Female	391	53.27
Grade	7	260	35.42
	8	307	41.83
	9	167	22.75
Family residence	Rural	474	64.58
	Urban	260	35.42
Only child or not	Yes	207	28.20
	No	527	71.80
Academic performance	Excellent	73	9.95
	Good	159	21.66
	Average	267	36.37
	Fair	176	23.98
	Poor	59	8.04
Parenting style	Harsh and tough	175	23.85
	Democratic and respectful	523	71.25
	Negligent and permissive	36	4.90

Table 2 shows the mean, standard deviation, range, and correlation values for all variables that include inferiority, fear of negative evaluation, social anxiety, and their factors. There were significant positive correlations between all factors and variables (*r* ≥ 0.21, *p* ≤ 0.001).

**Table 2:** Correlation matrix among variables (*n* = 734).

	1	2	3	4	5	6	7	8	9	10
1. Self-esteem	1									
2. Social confidence	0.60***	1								
3. Academic ability	0.57***	0.64***	1							
4. Appearance	0.51***	0.57***	0.46***	1						
5. Physical ability	0.43***	0.56***	0.53***	0.57***	1					
6. Inferiority	0.77***	0.90***	0.79***	0.74***	0.76***	1				
7. Social avoidance	0.44***	0.40***	0.32***	0.23***	0.28***	0.43***	1			
8. Social distress	0.38***	0.52***	0.36***	0.31***	0.35***	0.50***	0.67***	1		
9. Social anxiety	0.45***	0.50***	0.37***	0.30***	0.34***	0.51***	0.91***	0.92***	1	
10. FNE	0.41***	0.54***	0.38***	0.47***	0.38***	0.55***	0.21***	0.31***	0.29***	1
*M*	2.51	2.49	2.71	2.58	2.40	2.54	0.43	0.48	0.46	3.09
*SD*	0.74	0.79	0.65	0.74	1.04	0.63	0.23	0.25	0.22	0.73
Range	1.0–5.0	1.0–4.9	1.0–5.0	1.0–5.0	1.0–5.0	1.0–4.7	0–1.0	0–1.0	0–1.0	1.0–5.0

Note: FNE = fear of negative evaluation.

*** *p* < 0.001.

### 3.3. Examination of the mediation model

**Figure 2:**
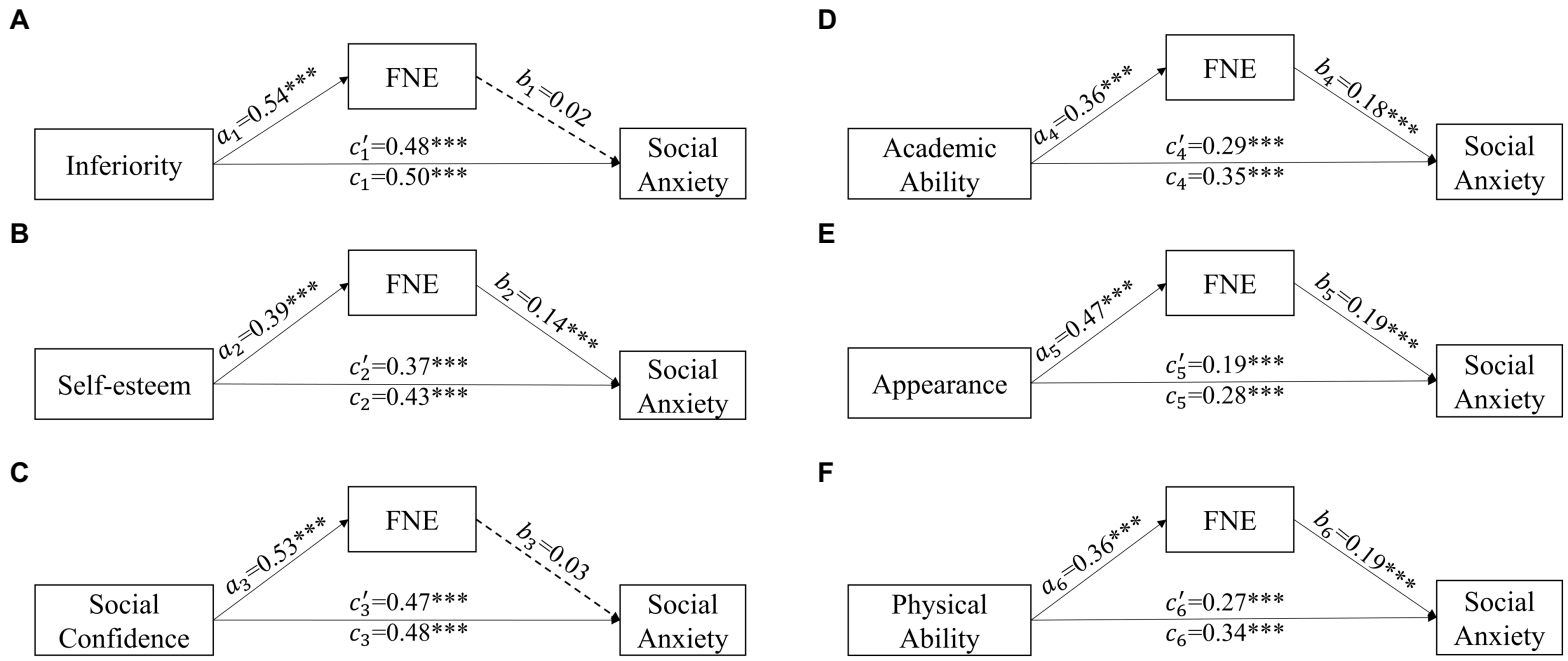
The mediation model of fear of negative evaluation on the relationship between inferiority and social anxiety.

This study used inferiority feelings as the independent variable, fear of negative evaluation as the mediating variable, and social anxiety as the dependent variable. The possible effect was tested by mediation analysis after controlling for demographic variables (age, gender, grade, family residence, only child situation, academic performance, and parenting style; see Figure 2; Tables 3, 4). The results of the mediation test showed that, in Model A, feelings of inferiority significantly and positively predicted fear of negative evaluation (β = 0.54, *p* < 0.001) and social anxiety (β = 0.48, *p* < 0.001). However, fear of negative evaluation’s prediction on social anxiety was not significant (β = 0.02, *p* > 0.05). Additionally, the bootstrap results for the mediating effect showed that the 95% confidence interval was [−0.03, 0.05] including 0, which indicates that the total score of fear of negative evaluation is not mediator in the predictive effect of total feelings of inferiority on social anxiety.

**Table 3:** Mediation effect of FNE on the relationship between inferiority and social anxiety.

Model	Outcome variable	Predictors	*R*	*R* ^2^	*SE*	*F*	*β*	*t*
Model A	Social anxiety	Inferiority	0.54	0.29	0.04	36.39***	0.50	15.53***
	FNE	Inferiority	0.57	0.33	0.36	44.09***	0.54	17.48***
	Social anxiety	Inferiority	0.54	0.29	0.04	32.35***	0.48	12.73***
		FNE					0.02	0.25
Model B	Social anxiety	Self-esteem	0.47	0.22	0.04	25.93***	0.43	12.71***
	FNE	Self-esteem	0.39	0.19	0.44	21.21***	0.39	11.42***
	Social anxiety	Self-esteem	0.49	0.24	0.04	25.21***	0.37	10.29***
		FNE					0.14	3.92***
Model C	Social anxiety	Social confidence	0.53	0.28	0.04	35.50***	0.48	15.31***
	FNE	Social confidence	0.56	0.32	0.37	41.80***	0.53	16.97***
	Social anxiety	Social confidence	0.53	0.28	0.04	31.62***	0.47	12.50***
		FNE					0.03	0.84
Model D	Social anxiety	Academic ability	0.41	0.17	0.04	18.47***	0.35	10.24***
	FNE	Academic ability	0.41	0.17	0.45	18.65***	0.36	10.53***
	Social anxiety	Academic ability	0.44	0.20	0.04	19.66***	0.29	7.89***
		FNE					0.18	4.94***
Model E	Social anxiety	Appearance	0.36	0.13	0.04	13.28***	0.28	8.09***
	FNE	Appearance	0.51	0.26	0.40	31.57***	0.47	14.48***
	Social anxiety	Appearance	0.39	0.16	0.04	14.81***	0.19	4.92***
		FNE					0.19	4.87***
Model F	Social anxiety	Physical ability	0.40	0.16	0.04	17.54***	0.34	9.89***
	FNE	Physical ability	0.41	0.17	0.45	18.63***	0.36	10.53***
	Social anxiety	Physical ability	0.44	0.19	0.04	18.94***	0.27	7.53***
		FNE					0.19	5.04***

Note: FNE = fear of negative evaluation.

*** *p* < 0.001.

**Table 4:** Bootstrap test for indirect effects.

Path	Effect	*SE*	LLCI	ULCI
Inferiority → FNE → social anxiety	0.01	0.02	−0.03	0.05
Self-esteem → FNE → social anxiety	0.06	0.02	0.03	0.09
Social confidence → FNE → social anxiety	0.02	0.02	−0.02	0.06
Academic ability → FNE → social anxiety	0.07	0.02	0.03	0.10
Appearance → FNE → social anxiety	0.09	0.02	0.05	0.13
Physical ability → FNE → social anxiety	0.07	0.02	0.04	0.10

Note: FNE = fear of negative evaluation; LLCI, low limit of confidence interval; ULCI, upper limit of confidence interval.

In current study, we took the five dimensions of inferiority (i.e., self-esteem, social confidence, academic ability, appearance, and physical ability) as independent variables, fear of unfavourable evaluation as mediating variables, and social anxiety as dependent variables. The results of the mediation test for the five models are described below (see Figure 2; Tables 3, 4).

In Model B, self-esteem significantly and positively predicted fear of negative evaluation (β = 0.39, *p* < 0.001) and social anxiety (β = 0.37, *p* < 0.001), and fear of negative evaluation significantly and positively predicted social anxiety (β = 0.14, *p* < 0.001). The bootstrapping test for indirect effect results showed that the mediating effect was 0.06, and the 95% confidence interval was [0.03, 0.09] excluding 0. These results indicate that fear of negative evaluation plays a partial mediating role in the relationship between self-esteem and social anxiety.

In Model C, social confidence significantly and positively predicted fear of negative evaluation (β = 0.53, *p* < 0.001) and social anxiety (β = 0.47, *p* < 0.001). However, the prediction of fear of unfavourable evaluation on social anxiety was not significant (β = 0.03, *p* > 0.05), and the bootstrap test results of the mediating effect showed that the 95% confidence interval was [−0.02, 0.06] including 0, which indicates that fear of negative evaluation does not play a mediating role in the relationship between social confidence and social anxiety.

In Model D, academic ability significantly and positively predicted fear of negative evaluation (β = 0.36, *p* < 0.001) and social anxiety (β = 0.29, *p* < 0.001), and fear of negative evaluation significantly and positively predicted social anxiety (β = 0.18, *p* < 0.001). The indirect effect bootstrapping test results showed that the mediating effect was 0.07. The 95% confidence interval was [0.03, 0.10] excluding 0, which indicates that fear of negative evaluation plays a partial mediating role in the relationship between academic ability and social anxiety.

In Model E, appearance significantly and positively predicted fear of negative evaluation (β = 0.47, *p* < 0.001) and social anxiety (β = 0.19, *p* < 0.001), and fear of negative evaluation significantly and positively predicted social anxiety (β = 0.19, *p* < 0.001). The indirect effect bootstrapping test results showed that the mediating effect was 0.09. The 95% confidence interval was [0.05, 0.13] excluding 0. These results indicate that fear of negative evaluation plays a partial mediating role in the relationship between appearance and social anxiety.

In Model F, physical ability significantly and positively predicted fear of negative evaluation (β = 0.36, *p* < 0.001) and social anxiety (β = 0.27, *p* < 0.001). Fear of unfavourable evaluation significantly and positively predicted social anxiety (β = 0.19, *p* < 0.001). The indirect effect bootstrapping test results showed that the mediating effect was 0.07. The 95% confidence interval was [0.04, 0.10] excluding 0, which indicates that fear of negative evaluation plays a partial mediating role in the relationship between physical ability and social anxiety.

## 4. Discussion

The present study investigated the relationship among feelings of inferiority, fear of negative evaluation, and social anxiety. We found that the subscales of these three variables were all significantly positively correlated. In the mediation analysis, fear of negative evaluation was a predictive mediator of the inferiority feelings of self-esteem, academic ability, appearance, and physical ability on social anxiety, but not the overall inferiority score and that of its subscale, social confidence.

The total score and each inferiority dimension of junior high school students had a significant positive correlation with social anxiety. These findings confirmed our Hypothesis 1 and were consistent with several studies (Payam and Agdasi, 2017), which suggested that the stronger the sense of inferiority, the greater the level of social anxiety. Students who feel inferior perform poorly in many aspects of everyday and academic life. They are afraid of being rejected and are afraid to interact with others. At the same time, they often use avoidance methods, which will exacerbate social anxiety (Yu and Liu, 2020).

The total score and each dimension of the inferiority feelings of junior high school students showed significant positive correlations with fear of negative evaluation. The higher the score of FIS, the stronger the fear of negative evaluation. These findings concur with previous studies (Geukens et al., 2020), indicating that adolescents with low self-esteem have greater fear of unfavorable evaluation by others. Fear of negative evaluation is a form of social anxiety. Adolescent junior high school students have strong egocentric characteristics. Often, they feel as if they are being observed by some imaginary audiences (Neff, 2003). Another study found that individuals with low self-esteem and low self-assessment are more afraid of negative evaluations (Borecka-Biernat, 2020). Therefore, students with higher inferiority feelings are not only characterized by low self-evaluation, but are also very afraid of negative evaluations by others.

There was a significant positive correlation between social anxiety and fear of negative evaluation of junior high school students, that is, the higher the score of the students’ social anxiety questionnaire, the stronger the performance of fear of negative evaluation, and vice versa. This result is consistent with that reported by Ajmal and Iqbal (Ajmal and Iqbal, 2019), revealing a positive correlation between social anxiety and fear of negative evaluation. The study concludes that fear of negative evaluation produces social anxiety in university students. Their research results align with those obtained in our study’s sample involving middle school students (Pan et al., 2018).

Further analysis showed that the mediating effect of fear of negative evaluation between the total inferiority score and social anxiety was not significant. In contrast, fear of unfavorable evaluation had a significant mediating effect between self-esteem, academic ability, appearance and physical ability and social anxiety. These findings partly support our Hypothesis 2. The exception to this mediating effect was social confidence. The reason is probably that the predictive effect of social confidence on social anxiety was higher than other four dimensions and fear of negative evaluation. In addition, confidence and anxiety are two opposite emotional experience in social interaction (O’Toole et al., 2013). Building confidence can effectively reduce social anxiety (Damer et al., 2010). Social confidence’s predictive effect on social anxiety may be less affected by fear of negative evaluation. Consequently, the mediating effect of fear of negative evaluation between the total inferiority score and social anxiety may be also significantly affected. However, a partial mediation existed in this study. Our results showed that the feelings of inferiority affecting junior high school students in self-esteem (Jiang and Ngien, 2020), academic ability (Strahan, 2003), appearance (Titchener and Wong, 2015), and physical ability (Dimech and Seiler, 2010) could directly predict their level of social anxiety status. In addition, it predicts their social anxiety status, which is mediated by fear of unfavorable evaluation. These results can be explained as follows. Some researchers indicate that fear of negative evaluation is the most common among young people, because negative evaluation can bring about comprehensive discomfort including embarrassment, anxiety, lack of ability, and feelings of inferiority (Ajmal and Iqbal, 2019). Students with inferiority in different dimensions are characterized by low self-concept and involuntarily accept the unfavorable evaluation of others (Murad, 2020). Hence, the students are not good at letting go from the emotional level and rationalizing from the cognitive level, which eventually generates anxiety and manifests higher levels of social anxiety.

Cognitive behavioral therapy promotes rational analysis and logical thinking to change the irrational beliefs of patients, and help them solve emotional and behavioral problems (Amin et al., 2020). Some studies have found that individuals with high levels of cognitive emotion regulation, fear of negative evaluation has a stronger predictive effect on social anxiety (Hong and Hong, 2011). It is a good intervention direction of CBT to know whether students with the feelings of inferiority in academic performance, appearance, physical condition and low self-esteem will have social anxiety, which can effectively reduce the degree of social anxiety. Therefore, junior high school students can reduce the degree of fear of negative evaluation by regulating cognitive emotion and reduce the effect of Inferiority in different dimensions on social anxiety.

The findings of this study may offer a reference value to prevent and reduce social anxiety among junior high school students in China. This study focuses on the prediction of social anxiety by specific and explicit inferiority feelings, such as self-esteem, academic ability, appearance, and physical ability. Chinese junior high schools and parents can base on the present findings to identify the initial underlying mind and behavior of students who suffer from social anxiety as a result. Schools can also reduce students’ social anxiety by teaching reasonable cognitive models and rectifying irrational beliefs about the fear of evaluation through the work of mental health education.

There are two limitations in this study. First, this study used a cross-sectional research design, and future studies can further explore a longitudinal design and cross-lagged analysis. In addition, the participants in this study were from the same region. Future studies are necessary to conduct across regions or across urban and rural areas.

## 5. Conclusion

The inferiority of self-esteem, academic ability, appearance, and physical ability may directly and indirectly predict social anxiety through the fear of negative evaluation. This study provides an important direction for social anxiety intervention in individuals with high scores of the inferiority in self-esteem, academic ability, appearance, and physical ability.

## Data availability statement

The raw data supporting the conclusions of this article will be made available by the authors, without undue reservation.

## Ethics statement

The studies involving human participants were reviewed and approved by the Ethics Committee of Minnan Normal University. Written informed consent to participate in this study was provided by the participants’ legal guardian/next of kin.

## Author contributions

JL and SC: conceptualization. JL and LW: methodology and writing—original draft. JL, SJ, MZ, and SC: validation and writing—review and editing. JL, SJ, and MZ: formal analysis and visualization. JL: investigation. JL and MZ: data curation. MZ and SC: supervision. SC: project administration and funding acquisition. All authors contributed to the article and approved the submitted version.

## Funding

This research was funded by the Fujian Province “Fourteenth Five-Year Plan” Education Science Planning Project (FJJKBK21-014).

## Conflict of interest

The authors declare that the research was conducted in the absence of any commercial or financial relationships that could be construed as a potential conflict of interest.

## Publisher’s note

All claims expressed in this article are solely those of the authors and do not necessarily represent those of their affiliated organizations, or those of the publisher, the editors and the reviewers. Any product that may be evaluated in this article, or claim that may be made by its manufacturer, is not guaranteed or endorsed by the publisher.

## References

[ref1] Aderka I. M. Hofmann S. G. Nickerson A. Hermesh H. Gilboa-Schechtman E. Marom S. (2012). Functional impairment in social anxiety disorder. J. Anxiety Disord. 26, 393–400. doi: 10.1016/j.janxdis.2012.01.003 22306132

[ref2] Adler A. (1927). Individual psychology. J. Abnorm. Soc. Psychol. 22, 116–122. doi: 10.1037/h0072190

[ref3] Agbaria Q. Ronen T. Hamama L. (2012). The link between developmental components (age and gender), need to belong and resources of self-control and feelings of happiness, and frequency of symptoms among Arab adolescents in Israel. Child Youth Serv. Rev. 34, 2018–2027. doi: 10.1016/j.childyouth.2012.03.009

[ref4] Ahadzadeh A. S. Rafik-Galea S. Alavi M. Amini M. (2018). Relationship between body mass index, body image, and fear of negative evaluation: moderating role of self-esteem. Health Psychol. Open 5:2055102918774251. doi: 10.1177/2055102918774251, PMID: 29977587 PMC6024295

[ref5] Ajmal A. Iqbal A. (2019). Fear of negative evaluation and social anxiety in young adults. Peshawar J. Psychol. Behav. Sci. 4, 45–53. doi: 10.32879/picp.2018.4.1.45

[ref6] Amin R. Iqbal A. Naeem F. Irfan M. (2020). Effectiveness of a culturally adapted cognitive behavioural therapy-based guided self-help (CACBT-GSH) intervention to reduce social anxiety and enhance self-esteem in adolescents: a randomized controlled trial from Pakistan. Behav. Cogn. Psychother. 48, 503–514. doi: 10.1017/S1352465820000284, PMID: 32450939

[ref7] Borecka-Biernat D. (2020). Adolescent coping strategies in social conflict in relation to self-esteem and cognitive appraisal of a conflict. Psychol. Rozwojowa 25, 31–48. doi: 10.4467/20843879PR.20.002.11999

[ref8] Chen Z. Y. (2002). Correlation between fear of negative evaluation and test anxiety among middle school students (in Chinese). Chin. Ment. Health J. 16, 855–857. doi: 10.3321/j.issn:1000-6729.2002.12.020

[ref9] Cheng Z. Binrong D. (2016). Relationship of fear of negative evaluation and social anxiety in college students (in Chinese). Chin. J. Health. Psychol. 11:039. doi: 10.13342/j.cnki.cjhp.2016.11.038

[ref10] Chi X. Hong X. Chen X. (2020). Profiles and sociodemographic correlates of internet addiction in early adolescents in southern China. Addict. Behav. 106:106385. doi: 10.1016/j.addbeh.2020.106385, PMID: 32187574

[ref11] Cimsir E. (2019). The roles of dispositional rumination, inferiority feelings and gender in interpersonal rumination experiences of college students. J. Gen. Psychol. 146, 217–233. doi: 10.1080/00221309.2018.1553844, PMID: 30663531

[ref12] Clark D. M. Wells A. (1995). “A cognitive model” in Social Phobia: Diagnosis, Assessment, and Treatment. ed. Heimberg R. G. (New York, NY, USA: The Guilford Press), 69–93.

[ref13] Collins R. L. (1996). For better or worse: the impact of upward social comparison on self-evaluations. Psychol. Bull. 119, 51–69. doi: 10.1037/0033-2909.119.1.51

[ref14] Damer D. E. Latimer K. M. Porter S. H. (2010). “Build your social confidence”: a social anxiety Group for College Students. J. Spec. Group Work 35, 7–22. doi: 10.1080/01933920903463510

[ref15] Dimech A. S. Seiler R. (2010). The association between extra-curricular sport participation and social anxiety symptoms in children. J. Clin. Sport Psychol. 4, 191–203. doi: 10.1123/jcsp.4.3.191

[ref16] Ergun-Basak B. Aydin M. (2019). Problematic internet use in terms of the purposes of internet use, irrational beliefs, feelings of inferiority, and gender. Addicta: the Turkish. J. Addict. 6, 469–494. doi: 10.15805/addicta.2019.6.3.0017

[ref17] Fleming J. S. Courtney B. E. (1984). The dimensionality of self-esteem: II. Hierarchical facet model for revised measurement scales. J. Pers. Soc. Psychol. 46, 404–421. doi: 10.1037/0022-3514.46.2.404

[ref18] Geukens F. Maes M. Spithoven A. Pouwels J. L. Danneel S. Cillessen A. H. N.. (2020). Changes in adolescent loneliness and concomitant changes in fear of negative evaluation and self-esteem. Int. J. Behav. Dev. 46, 10–17. doi: 10.1177/0165025420958194

[ref19] Haikal M. Hong R. Y. (2010). The effects of social evaluation and looming threat on self-attentional biases and social anxiety. J. Anxiety Disord. 24, 345–352. doi: 10.1016/j.janxdis.2010.01.007, PMID: 20176459

[ref20] Hayes A. F. (2017). Introduction to Mediation, Moderation, and Conditional Process Analysis, Second Edition: A Regression-Based Approach. New York: Guilford Publications.

[ref21] Hong K. Hong H. (2011). The effects of fear of negative evaluation and cognitive emotional regulation on adolescents’ social anxiety. Korean J. Youth Stud. 18, 291–319. doi: 10.5762/KAIS.2015.16.10.6895

[ref22] Jiang S. Ngien A. (2020). The effects of Instagram use, social comparison, and self-esteem on social anxiety: a survey study in Singapore. Soc. Media Soc. 6:205630512091248. doi: 10.1177/2056305120912488

[ref23] Kalin N. H. (2020). The critical relationship between anxiety and depression. Am. J. Psychiatry 177, 365–367. doi: 10.1176/appi.ajp.2020.20030305 32354270

[ref24] Leary M. R. (1983). A brief version of the fear of negative evaluation scale. Personal. Soc. Psychol. Bull. 9, 371–375. doi: 10.1177/0146167283093007

[ref25] Li J. Li J. Jia R. Wang Y. Qian S. Xu Y. (2020). Mental health problems and associated school interpersonal relationships among adolescents in China: a cross-sectional study. Child Adolesc. Psychiatry Ment. Health 14:12. doi: 10.1186/s13034-020-00318-6, PMID: 32256690 PMC7106742

[ref26] Li S. Wang S. Gao X. Jiang Z. Xu H. Zhang S.. (2021). Patterns of adverse childhood experiences and suicidal behaviors in adolescents: a four-province study in China. J. Affect. Disord. 285, 69–76. doi: 10.1016/j.jad.2021.02.045, PMID: 33636673

[ref27] Lin Y. Fan Z. (2022). The relationship between rejection sensitivity and social anxiety among Chinese college students: the mediating roles of loneliness and self-esteem. Curr. Psychol., 1–10. doi: 10.1007/s12144-021-02443-7

[ref28] Liu L. Chen X.-L. Ni C.-P. Yang P. Huang Y.-Q. Liu Z.-R.. (2018b). Survey on the use of mental health services and help-seeking behaviors in a community population in northwestern China. Psychiatry Res. 262, 135–140. doi: 10.1016/j.psychres.2018.02.010, PMID: 29433108

[ref29] Liu H. Shi Y. Auden E. Rozelle S. (2018a). Anxiety in rural Chinese children and adolescents: comparisons across provinces and among subgroups. Int. J. Environ. Res. Public Health 15:2087. doi: 10.3390/ijerph15102087, PMID: 30248994 PMC6210330

[ref30] Liu Y. Xu C. Kuai X. Deng H. Wang K. Luo Q. (2022). Analysis of the causes of inferiority feelings based on social media data with Word2Vec. Sci. Rep. 12, 5218–5219. doi: 10.1038/s41598-022-09075-2, PMID: 35338206 PMC8956725

[ref31] Lyu L. (2022). “The cause and solution towards Chinese adolescents inferior”, in: 2021 international conference on education, language and art (ICELA 2021): Atlantis Press. pp. 43–48.

[ref32] Morrison A. S. Heimberg R. G. (2013). Social anxiety and social anxiety disorder. Annu. Rev. Clin. Psychol. 9, 249–274. doi: 10.1146/annurev-clinpsy-050212-185631 23537485

[ref33] Morrissette M. (2021). School closures and social anxiety during the COVID-19 pandemic. J. Am. Acad. Child Adolesc. Psychiatry 60, 6–7. doi: 10.1016/j.jaac.2020.08.436, PMID: 32890669 PMC7467010

[ref34] Murad O. S. (2020). Social anxiety in relation to self-esteem among university students in Jordan. Int. Educ. Stud. 13:96. doi: 10.5539/ies.v13n2p96

[ref35] Neff K. (2003). Self-compassion: an alternative conceptualization of a healthy attitude toward oneself. Self Identity 2, 85–101. doi: 10.1080/15298860309032

[ref36] Nordahl H. Plummer A. Wells A. (2017). Predictors of biased self-perception in individuals with high social anxiety: the effect of self-consciousness in the private and public self domains. Front. Psychol. 8:1126. doi: 10.3389/fpsyg.2017.01126, PMID: 28725207 PMC5495823

[ref37] O’Toole M. S. Hougaard E. Mennin D. S. (2013). Social anxiety and emotion knowledge: a meta-analysis. J. Anxiety Disord. 27, 98–108. doi: 10.1016/j.janxdis.2012.09.005 23247206

[ref38] Pan Z. Zhang D. Liu G. Luo S. (2018). The mediating role of fear of evaluation between psychological Suzhi and social anxiety among Chinese secondary school students. Curr. Psychol. 38, 1174–1181. doi: 10.1007/s12144-018-0009-2

[ref39] Payam R. Agdasi A. (2017). The relationship between the mental health and the inferiority complex with social anxiety amongfemale students. Woman Stud. Fam. 9, 7–27.

[ref40] Podsakoff N. P. MacKenzie S. B. Lee J.-Y. Podsakoff N. P. (2003). Common method biases in behavioral research: A critical review of the literature and recommended remedies. J. Appl. Psychol. 88, 879–903. doi: 10.1037/0021-9010.88.5.879 14516251

[ref41] Portillo M. Fernández-Baena J. (2019). Social self-perception in adolescents: accuracy and bias in their perceptions of acceptance/rejection. Educ. Psychol. 26, 1–6. doi: 10.5093/psed2019a12

[ref42] Rapee R. M. Heimberg R. G. (1997). A cognitive-behavioral model of anxiety in social phobia. Behav. Res. Ther. 35, 741–756. doi: 10.1016/S0005-7967(97)00022-3 9256517

[ref43] Schunk F. Wong N. Nakao G. Trommsdorff G. (2022). Different functions of emotion regulation in linking harmony seeking and rejection avoidance to life satisfaction and social support in Germany, Hong Kong, and Japan. Asian J. Soc. Psychol. doi: 10.1111/ajsp.12557

[ref44] Shim S. S. Wang C. Cassady J. C. (2013). Emotional well-being: the role of social achievement goals and self-esteem. Personal. Individ. Differ. 55, 840–845. doi: 10.1016/j.paid.2013.07.004

[ref45] Stein M. B. Jang K. L. Livesley W. J. (2002). Heritability of social anxiety-related concerns and personality characteristics: a twin study. J. Nerv. Ment. Dis. 190, 219–224. doi: 10.1097/00005053-200204000-00002, PMID: 11960082

[ref46] Strahan E. Y. (2003). The effects of social anxiety and social skills on academic performance. Personal. Individ. Differ. 34, 347–366. doi: 10.1016/S0191-8869(02)00049-1

[ref47] Sun J. Dunne M. P. Hou X. (2012). Academic stress among adolescents in China. Aust. Epidemiol. 19, 9–12.

[ref48] Tang S. (2012). A review of researches on inferiority complex at home and abroad (in Chinese). Intelligence, 187–188.

[ref49] Tang D. Wen Z. (2020). Statistical approaches for testing common method bias: problems and suggestions (in Chinese). J. Psychol. Sci. 43, 215–223. doi: 10.16719/j.cnki.1671-6981.20200130

[ref50] Titchener K. Wong Q. J. (2015). A weighty issue: explaining the association between body mass index and appearance-based social anxiety. Eat. Behav. 16, 13–16. doi: 10.1016/j.eatbeh.2014.10.005, PMID: 25464060

[ref52] Wang X. D. Wang X. L. Ma H. (1999). Manual of mental health assessment scale, revised edition (in Chinese). Chin. Ment. Health J. 214–216.

[ref53] Watson D. Friend R. (1969). Measurement of social-evaluative anxiety. J. Consult. Clin. Psychol. 33, 448–457. doi: 10.1037/h0027806 5810590

[ref54] Wilkinson R. G. (1999). Health, hierarchy, and social anxiety. Ann. N. Y. Acad. Sci. 896, 48–63. doi: 10.1111/j.1749-6632.1999.tb08104.x 10681887

[ref55] Wu Z. Liu Z. Zou Z. Wang F. Zhu M. Zhang W.. (2021). Changes of psychotic-like experiences and their association with anxiety/depression among young adolescents before COVID-19 and after the lockdown in China. Schizophr. Res. 237, 40–46. doi: 10.1016/j.schres.2021.08.020, PMID: 34481204 PMC8437585

[ref56] Yamagishi T. Hashimoto H. Schug J. (2008). Preferences versus strategies as explanations for culture-specific behavior. Psychol. Sci. 19, 579–584. doi: 10.1111/j.1467-9280.2008.02126.x, PMID: 18578848

[ref57] You Z. Zhang Y. Zhang L. Xu Y. Chen X. (2019). How does self-esteem affect mobile phone addiction? The mediating role of social anxiety and interpersonal sensitivity. Psychiatry Res. 271, 526–531. doi: 10.1016/j.psychres.2018.12.040, PMID: 30553099

[ref58] Yu S. Liu Q. X. (2020). The effect of parental neglect on adolescent suicidal ideation: the mediating role of self-esteem and hope (in Chinese). Psychol. Dev. Educ. 36, 350–358. doi: 10.16187/j.cnki.issn1001-4918.2020.03.12

[ref59] Yücens B. Üzer A. (2018). The relationship between internet addiction, social anxiety, impulsivity, self-esteem, and depression in a sample of Turkish undergraduate medical students. Psychiatry Res. 267, 313–318. doi: 10.1016/j.psychres.2018.06.033, PMID: 29957547

[ref60] Zhong Y. J. Zhang J. F. (2011). The mediating effect of fear of evaluation on the relations between self-esteem and social anxiety for college students (in Chinese). J. Psychol. Dev. Educ. 5, 506–512. doi: 10.16187/j.cnki.issn1001-4918.2011.05.002

[ref61] Zhou S. J. Zhang L. G. Wang L. L. Guo Z. C. Wang J. Q. Chen J. C.. (2020). Prevalence and socio-demographic correlates of psychological health problems in Chinese adolescents during the outbreak of COVID-19. Eur. Child Adolesc. Psychiatry 29, 749–758. doi: 10.1007/s00787-020-01541-4, PMID: 32363492 PMC7196181

